# Supplementary material for: Analysis of PPARγ Signaling Activity in Psoriasis
Source: Int J Mol Sci. 2021 Aug 10;22(16):8603. doi: 10.3390/ijms22168603 (PMC8395241; doi:10.3390/ijms22168603)
Supplement: Supplementary file 1 [file ijms-22-08603-s001.zip › Supplemental materials_Analysis of PPARg signaling activity in psoriasis/Pathway models/Models images and html files/PPARG inhibits phenotypes assotiated with psoriasis/134191.html]

cirrhosis


# Disease cirrhosis

|  |  |
| --- | --- |
| URN | urn:agi-meshdis:Liver%20Cirrhosis |
| Total Entities | 8 |
| Connectivity | 5152 |
| Name | cirrhosis |

---

|  |  |
| --- | --- |
| ChildConcepts | Indian childhood cirrhosis |
|  | compensated liver cirrhosis |
|  | alcoholic cirrhosis |
|  | biliary cirrhosis |
|  | congenital hepatic fibrosis |
|  | hepatic decompensation |
|  | micronodular cirrhosis |
|  | primary biliary cirrhosis |

---

|  |  |
| --- | --- |
| Pathway | Argininosuccinic Aciduria in Urea Cycle Disorders |
|  | PPARG inhibits phenotypes assotiated with psoriasis |

---

|  |  |
| --- | --- |
| MedScan ID | 9003337 |

---

|  |  |
| --- | --- |
| Alias | Cirrhosis, Liver |
|  | Hepatic Cirrhosis |
|  | Cirrhoses, Liver |
|  | Cirrhosis, Hepatic |
|  | Liver Cirrhosis |
|  | cirrhotic livers |
|  | dietary cirrhosis |
|  | Hepatic Cirrhoses |
|  | cirrhotic liver |
|  | Cirrhosis |
|  | cirrhotic |
|  | Cirrhoses |
|  | postnecrotic liver cirrhosis |
|  | dietary liver cirrhosis |
|  | Liver Cirrhoses |
|  | postnecrotic cirrhosis of the liver |
|  | cirrhosis hepatis |
|  | cryptogenic liver cirrhosis |

---

|  |  |
| --- | --- |
| MeSH ID | D008103 |
|  | M0012636 |

---

|  |  |
| --- | --- |
| MeSH Heading | Liver Cirrhosis |

---
